# TNF-α inhibits SATB2 expression and osteoblast differentiation through NF-κB and MAPK pathways

**DOI:** 10.18632/oncotarget.23373

**Published:** 2017-12-18

**Authors:** Chijian Zuo, Xiaoying Zhao, Yu Shi, Wen Wu, Ning Zhang, Jiake Xu, Chuandong Wang, Guoli Hu, Xiaoling Zhang

**Affiliations:** ^1^ Department of Orthopedic Surgery, Xinhua Hospital, School of Medicine, Shanghai Jiao Tong University, Shanghai 200092, China; ^2^ The Key Laboratory of Stem Cell Biology, Shanghai Institutes for Biological Sciences, Chinese Academy of Sciences, Shanghai 200025, China; ^3^ Shanghai Key Laboratory of Orthopaedic Implant, Department of Orthopaedics, Shanghai Ninth People's Hospital, Shanghai Jiao Tong University School of Medicine, Shanghai 200011, China; ^4^ School of Pathology and Laboratory Medicine, University of Western Australia, Perth, Western Australia 6009, Australia

**Keywords:** TNF-α, SATB2 inhibition, osteoblast differentiation, inflammation-induced bone loss

## Abstract

Although the mechanisms of Tumor necrosis factor alpha (TNF-α) on facilitating osteoclast differentiation and bone resorption is well known, the mechanisms behind the suppression of the osteoblast differentiation from mesenchymal stem cells (MSCs) are still poorly understood. In this study, we observed a negative correlation between TNF-α levels and the expression of special AT-rich sequence-binding protein 2 (SATB2), a critical osteoblastogenesis transcription factor, in ovariectomy (OVX)-induced bone loss and IL-1-induced arthritis animal model. We found that TNF-α treatment inhibited mesenchymal cell line C2C12 osteoblast differentiation and sharply decreased BMP2-induced SATB2 expression. Upon TNF-α treatment, the activity of smad1/5/8 was inhibited, by contrast, extracellular signal-regulated kinase-1/2 (ERK1/2) and P38 was increased in C2C12 cells, the inhibitor of ERK1/2 (U0126) was found to abrogate the TNF-α inhibition of SATB2 expression. Furthermore, the NF-κB signaling pathway in C2C12 cells was significantly activated by the treatment of TNF-α, and TNF-α induced NF-κB directly binds to SATB2 promoter to suppress its expression. These results suggest that TNF-α suppresses SATB2 expression through activating NF-κB and MAPK signaling and depressing smad1/5/8 signaling, which contributes to the inhibition of osteoblast differentiation and might be potential therapeutic targets for inflammation-induced bone loss.

## INTRODUCTION

Pathological status such as high inflammatory responses often leads to an imbalance of bone remodeling which usually results in bone loss such as osteoporosis and arthritis [1–3]. Tumor necrosis factor alpha (TNF-α) is critical for the pathogenesis of bone loss as it stimulates osteoclastic bone resorption and suppresses osteoblastic bone formation. Although the mechanisms of TNF-α in facilitating osteoclast differentiation and bone resorption is well known, the mechanisms behind the suppression of the osteoblast differentiation from mesenchymal stem cells (MSCs) are still poorly understood. There are reports demonstrating that TNF-α inhibits osteoblast differentiation by suppressing the expression of the key regulators of osteoblast differentiation, Runt-related gene 2 (Runx2) [4, 5] and Osterix (Osx) [6]. TNF-α stimulates Wwp1 expression and facilitates the transcription factor JunB degradation, which in turn, attenuates MSCs osteoblast differentiation [7]. Moreover, TNF-α displays its inhibitory effect on osteoblastogenesis through repressing the BMP signal by interfering with the DNA binding of Smads through the activation of NF-κB [8]. TNF-α enhanced miR-3305p expression in BMP2-induced human BMSCs and miR-3305p targets SATB2 to suppress SATB2 expression, which, in turn, inhibiting BMP2-induced osteogenesis [9]. However, due to the complexity of the transcriptional network of osteoblast differentiation and the complex signaling of TNF-α and osteoblast-regulating factors, the above findings can only partially explain the molecular mechanisms of the inhibition of osteoblastogenesis by TNF-α.

Special AT-rich sequence-binding protein 2 (SATB2) was found to be a multifunctional determinant of craniofacial patterning and osteoblast differentiation in bone development regulation [10]. SATB2 is a transcription factor that belongs to the special AT-rich binding proteins family which binds to nuclear matrix-attachment regions (MARs), and activates transcription in a MAR-dependent manner [11]. SATB2 associates with Histone Deacetylase 1 (HDAC1) and Metastasis associated protein 2 (MAT2) to modify the chromatin structure so as to exert an important role in integrating genetic and epigenetic signals to modulate gene transcriptional activity [12, 13]. Moreover, SATB2 itself functions as a transcriptional factor to associate with other transcriptional factors such as Runx2 and ATF4 to activate or suppress its target genes expression [14]. Furthermore, lentival-mediated expression of SATB2 triggers osteogenic differentation of mouse BMSCs and promotes bone formation through a tissue-engineering technique [15]. The deficiency or haploinsufficiency of SATB2 in humans and mice is predicted to result in several severe diseases; including cleft palate, osteoporosis, craniofacial dysformation, craniosynostosis and mental retardation [10, 14, 16]. Moreover, SATB2 protects osteoblasts against oxidative stress-induced apoptotic insults [17]. Intriguingly, SATB2 was documented to be a sensitive marker for bone tumors, especially osteosarcoma [18–20], which might serve as a practically, diagnostically or prognostically useful marker for bone tumors. These accumulating evidences substantiate that SATB2 serves a pivotal role in skeletal development and osteoblastic differentiation.

Although the function and molecular mechanisms of SATB2 in regulating embryonic bone formation and osteoblast differentiation have been elucidated, the manner by which extracellular signals and other transcription factors regulate *Satb2* gene expression remains to be explored. It was found that BMP2/4/7 proteins enhance SATB2 expression by activating smad1/5, and smad1/5 directly interacts with SATB2 gene promoter to promote its expression [21]. Last but not the least, several micro- RNAs that targets SATB2 were reported to be involved in modulation of SATB2 expression: in BMSC osteo-induction process, miR-205 could significantly influence the expression of SATB2 to regulate osteoblast differentiation [22]; in mice miR-34b/c targets SATB2 to inhibit osteoblast proliferation and differentiation [23]. However, to date, information on the regulation of *Satb2* gene expression by inflammatory factors is limited.

The present study explores the regulation of *Satb2* gene expression by critical inflammatory cytokine TNF-α. We found that the SATB2 expression levels were negatively associated with the expression levels of TNF-α both in ovariectomy (OVX) -induced bone loss and IL-1β-induced arthritis animal models. Using mesenchymal cell line C2C12 osteoblast differentiation model, we confirmed that BMP2 stimulates SATB2 expression and this up-regulation could be significantly inhibited by TNF-α in a concentration-dependent and durable manner. To further understand the mechanism of TNF-α suppression on SATB2, smad1/5/8, mitogen-activated protein kinase (MAPK) and nuclear factor-κB (NF-κB) signaling pathways and their roles in the regulation of SATB2 expression were investigated in current study. Understanding the expression regulation of SATB2 by cell-extrinsic signals and inflammatory factors gives new insights into the mechanisms of the inhibition of inflammatory factors on osteoblast differentiation. Besides, these findings provide great significance to clinical treatment in inflammatory-induced osteoporosis and bone loss.

## RESULTS

### The expression level of SATB2 is negatively correlated with TNF-α level in OVX-induced bone loss and IL-1β-induced arthritis mice models

**Figure 1:**
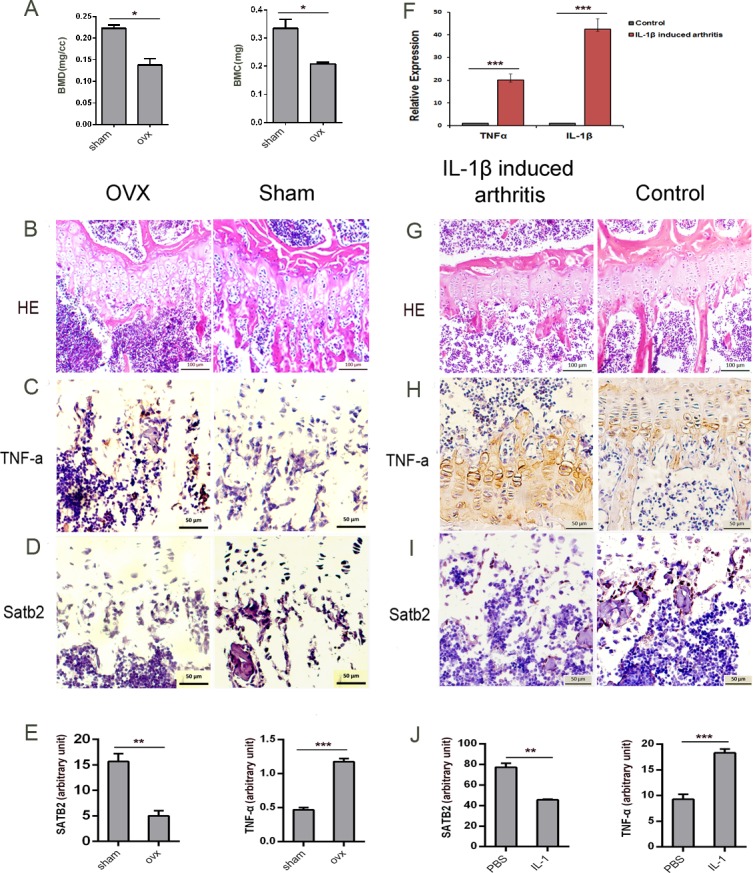
The expression level of SATB2 is negatively correlated with TNF-α level **(A)** Bone Mineral Density (BMD), Bone Mass Index (BMI) of femurs from OVX and sham-operated mice (*n* = 9) were assessed. **(B-D, G-I)** Femurs from OVX (B, C, D left) and sham-operated (B, C, D right) mice, IL-1β- induced arthritis mice (G, H, I left) and saline-induced control mice (G, H, I right) are embedded and sectioned to undergo H&E staining (B, G) and immunohistochemistry with TNF-α (C, H) and SATB2 (D, I) antibodies. **(E)** Density analysis of C and D. **(F)** TNF-α and IL-1β were detected by ELISA from IL-1β- induced arthritis mice and saline-induced control mice synovia. **(J)** Density analysis of H and I. The data are presented as mean ± S.D. (*n* = 9; ^*^p < 0.05; ^**^p < 0.01; ^***^p < 0.001).

In ovariectomy (OVX) -induced bone loss and IL-1β-induced arthritis mice models, we examined SATB2 and TNF-α expression levels by immunohistochemistry using the antibodies specific for TNF-α and SATB2. To proof the models are successful, the BMD and BMC of the OVX- and sham-operated mice were examined using micro-CT (Figure 1A) and the bone mass were shown by H & E staining (Figure 1B) and the levels of TNF-α and IL-1β in the synovia in the IL-1β-induced arthritis mice and PBS-induced control mice were detected by ELISA (Figure 1F) and the bone mass were shown by H & E staining (Figure 1G). TNF-α expression (Figure 1C, 1E) was higher in OVX-induced mice bone than that in sham-operated mice, which is consistent with previous reports [24], by contrast, SATB2 expression was less in the osteoblasts both in the growth plate and in the bone lining cells of bone trabecula in OVX mice than that in sham-operated mice (Figure 1D, 1E). In IL-1β-induced arthritis mice, there were intense staining of TNF-α (Figure 1H left, 1J) but weak staining of SATB2 (Figure 1I left, 1J) in mature osteoblasts. However, in PBS treated control mice, TNF-α (Figure 1H right, 1J) was moderately expressed and SATB2 (Figure 1I right, 1J) was intensely expressed in mature osteoblasts. As demonstrated above, SATB2 expression levels were negatively associated with the levels of TNF-α both in OVX-induced bone loss and IL-1β-induced arthritis mice. These observations indicated that TNF-α might negatively regulate SATB2 expression during osteoblastogenesis and bone formation, and thus inhibit bone formation.

### TNF-α inhibits osteoblastogenesis and SATB2 expression

To determine whether TNF-α inhibits SATB2 expression during osteoblast differentiation and bone formation, we constructed an osteogenic differentiation model by inducing the mesenchymal cell line C2C12 to differentiate into osteoblasts with Adenovirus-BMP2 (Adv-BMP2). Adv-BMP2 is reconstructed adenovirus that contains the human BMP2 expressing sequence in the adenovirus vector. In the current study, the C2C12 cells were treated with Adv-β-Gal (150 pfu/cell) or Adv-BMP2 (150 pfu/cell) for five days and followed by measurement of osteoblast differentiation. The quantitative real-time PCR indicates that the Adv-BMP2 group had significantly higher expression levels of osteoblast-specific gene *collagen I (Col I), bone sialoprotein (Bsp)*, and *osteocalcin (Ocn)* (Supplementary Figure 1A). Furthermore, *alkaline phosphatase (ALP*), another osteoblast-specific marker gene, was significantly increased in the Adv-BMP2 group, as shown by the ALP activity assay (Supplementary Figure 1A) and staining tests (Supplementary Figure 1B). These results suggest that the C2C12 cells could be induced to undergo osteoblastic differentiation through Adv-BMP2 stimulation in the present system.

**Figure 2:**
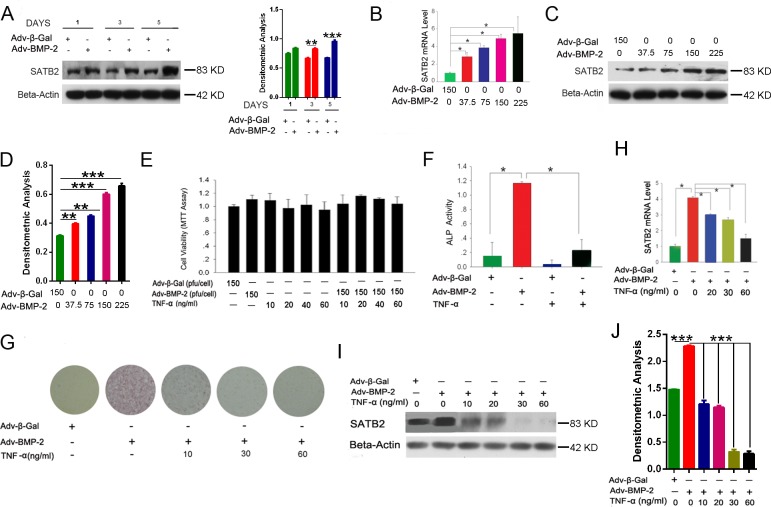
TNF-α inhibits osteoblastogenesis and SATB2 expression **(A)** The C2C12 cells were treated with Adv-BMP2 (150 pfu/cell) or Adv-β-Gal (150 pfu/cell) for one, three, and five days and SATB2 expression was examined by western blot and then underwent densitometric analysis. **(B, C, D)** The C2C12 cells were treated with Adv-β-Gal (150 pfu/cell) or various concentrations of Adv-BMP2 for three days. The SATB2 relative mRNA levels (B) and protein levels (C) were assessed and densitometric analysis of C (D) was graphed. **(E)** The C2C12 cells were treated with Adv-β-Gal (150 pfu/cell), Adv-BMP2 (150 pfu/cell), or 10 ng/mL to 60 ng/mL of TNF-α for 72 h, then followed by MTT assay. **(F)** The C2C12 cells were treated with Adv-β-Gal (150 pfu/cell), Adv-BMP2 (150 pfu/cell), or 60 ng/mL of TNF-α for five days. ALP activity were measured. **(G)** The C2C12 cells were treated with Adv-β-Gal (150 pfu/cell), Adv-BMP2 (150 pfu/cell), or 10 ng/mL to 60 ng/mL of TNF-α for five days and ALP staining was performed. **(H^_^J)** The C2C12 cells were treated with Adv-β-Gal (150 pfu/cell), Adv-BMP2 (150 pfu/cell), or 10 ng/mL to 60 ng/mL of TNF-α for 72 h. The *SATB2* gene expressions were assessed by real-time PCR (H) and western blot (I) and densitometric analysis (J). The data are presented as mean ± S.D. (*n* = 3; ^*^p < 0.05; ^**^p < 0.01; ^***^p < 0.001), these western blot images were uncropped.

Considering that SATB2 was unveiled to be a multifunctional determinant of osteoblast differentiation, we postulated that the expression level of SATB2 may consistently change during the osteogenesis of C2C12 cells. Supplementary Figure 1C shows that, the SATB2 mRNA level steadily increased from day 2 to day 5 under Adv-BMP2 stimulation and was significantly higher than that of the control group each day. Besides, the SATB2 protein was significantly increased by Adv-BMP2 stimulation after days 3 and 5 (Figure 2A). Furthermore, the C2C12 cells were treated with various concentrations of Adv-BMP2 (37.5 pfu/cell to 225 pfu/cell) during day 3 of the culturing process. The present results indicated that the SATB2 expression was up-regulated along with increased Adv-BMP2 concentrations (Figure 2B). Similar changes in the protein levels of SATB2 were found (Figure 2C and 2D). Taken together, these findings indicate that BMP2 stimulates SATB2 gene and protein expression in a time- and concentration-dependent manner.

Considering that TNF-α is well known for having the ability to induce cell apoptosis, first, the C2C12 cells were treated with several doses of TNF-α (10 ng/mL to 60 ng/mL) or combined with Adv-BMP2 (150 pfu/cell) for three days and then undergone with MTT assay. The results indicate that the relative cell viability rates at different treatments are not significantly different from those of the control group, suggesting that TNF-α (10 ng/mL to 60 ng/mL) has no significant toxic effect on C2C12 cells in our culturing system (Figure 2E). To determine whether TNF-α has an inhibitory effect on the osteoblast differentiation of C2C12 cells induced by Adv-BMP2, the C2C12 cells were treated with Adv-BMP2 (150 pfu/cell), TNF-α (60 ng/mL), or a combination of TNF-α (60 ng/mL) and Adv-BMP2 (150 pfu/cell) for five days and ALP activity was assayed. The results show that the cells treated with TNF-α had significant lower ALP activity compared with the control group (Figure 2F).

To determine whether the inhibition is concentration dependent, the C2C12 cells were treated with three different concentrations (10, 30, and 60 ng/mL) of TNF-α combined with Adv-BMP2 for five days and the osteoblast differentiation rates were detected by ALP staining. Figure 2G shows that the ALP positive rates decline with the elevated concentration of TNF-α treatment and even 10 ng/mL of TNF-α has a significant inhibition of ALP activity. The osteoblastic differentiation of C2C12 cells was totally blocked by high concentrations of TNF-α (30 ng/mL and 60 ng/mL). These results suggest that TNF-α significantly inhibits osteoblastic differentiation of C2C12 cells induced by Adv-BMP2 in a concentration-dependent manner and that the inhibitory effect was independent of cell apoptosis. Aiming to elucidate the molecular mechanism of the suppression of osteoblastogenesis by TNF-α, we further investigated the relationship between TNF-α and SATB2 expression in this system. The SATB2 expression was analyzed in C2C12 cells after TNF-α treatment using quantitative real-time PCR and Western blot method. Figure 2H, 2I and 2J show that the addition of TNF-α significantly suppressed SATB2 expression, which was stimulated by Adv-BMP2 (150 pfu/cell) in a three-day treatment. These results suggest that TNF-α inhibits SATB2 expression in a concentration-dependent manner, which is consistent to its inhibitory effect on osteoblast differentiation.

### Over-expression of SATB2 rescues TNF-α inhibition of osteoblastogenesis

**Figure 3:**
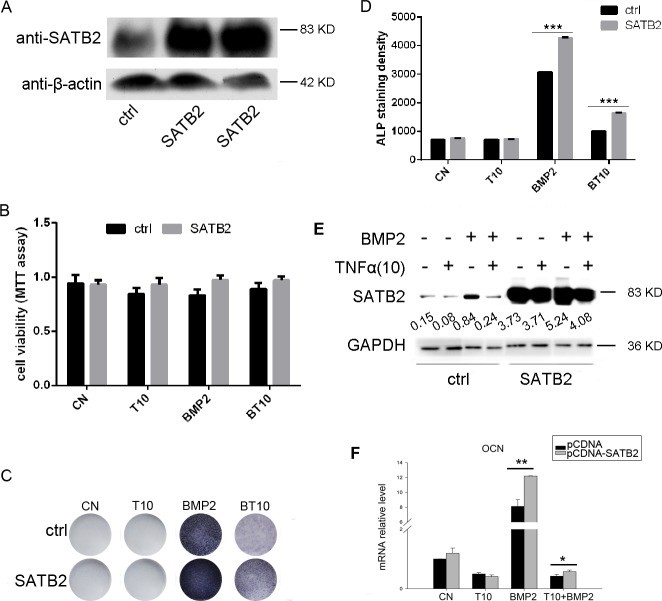
Over-expression of SATB2 rescues TNF-α inhibition of osteoblastogenesis **(A)** SATB2 over-expression lentivirus and control lentivirus were transfected into C2C12 cells to construct cell lines that over-express SATB2 constantly, two cell lines were chosen and uncropped western blot images were shown. **(B^_^E)** Control cell lines and SATB2 over-expression cell lines from (A) were treated with or without 250 ng/ml BMP2 and TNF-α as indicated for 3 days, followed by MTT assay (B) and ALP staining (C). The ALP staining density in figure B were analyzed with image J software (D) and the SATB2 expression levels were detected using western blotting and uncropped western blot images were shown (E). (D) C2C12 cells were transfected with pCDNA3.1+ vector or pCDNA3.1+-SATB2 vector and then treated with TNF-α or BMP2 (250ng/ml) as indicated for 5 days, the OCN mRNA levels were measured with real time RT-PCR. The data are presented as mean ± S.D. (*n=3,* ^*^p < 0.05; ^**^p < 0.01; ^***^p < 0.001, T10 means 10 ng/ml TNF-α, BT10 means 250 ng/ml BMP2 and 10 ng/ml TNF-α).

To further determine whether TNF-α inhibits osteoblast differentiation through inhibition of SATB2 expression and whether SATB2 serves as a vital factor in osteoblastogenesis, we constructed C2C12 cell lines that consistently over-express SATB2 with lenti-viruses. Figure 3A indicates that two strains of C2C12 cells significantly increase the basal level of SATB2 protein. C2C12 cells were treated with or without TNF-α and BMP2 as indicated in Figure 3B-3E for 3 days, followed by MTT assay, ALP staining and western blotting, Figure 3C and 3D show that TNF-α treatment reduces the ALP positive cells, however, over-expression of SATB2 enhances the staining density and rescues the reduction of TNF-α on the staining density. However, both TNF-α treatment and overexpression of SATB2 have no effect on C2C12 cell proliferation and TNF-α exert same effect on SATB2 expression both in control and SATB2-over-expression C2C12 cells (Figure 3B and 3E). We then over-expressed SATB2 by transfecting plasmid pCDNA3.1-SATB2 into C2C12 cells, and mRNA level of OCN, marker of terminal osteoblast differentiation, was examined under adv-BMP2 or TNF-α treatment. As shown in Figure 3F, over-expression of SATB2 enhanced and partly rescued TNF-α inhibition of the expression of OCN. As reported before, ALP and OCN are master genes in osteoblastic commitment during osteoblastogenesis, enhanced expression of ALP and OCN is in line with enhanced osteogenesis. These results indicated that over-expression of SATB2 rescued the inhibitory effect of TNF-α on osteoblastogenesis.

### TNF-α inhibits SATB2 expression by inhibiting the smad1/5/8 signaling pathway

**Figure 4:**
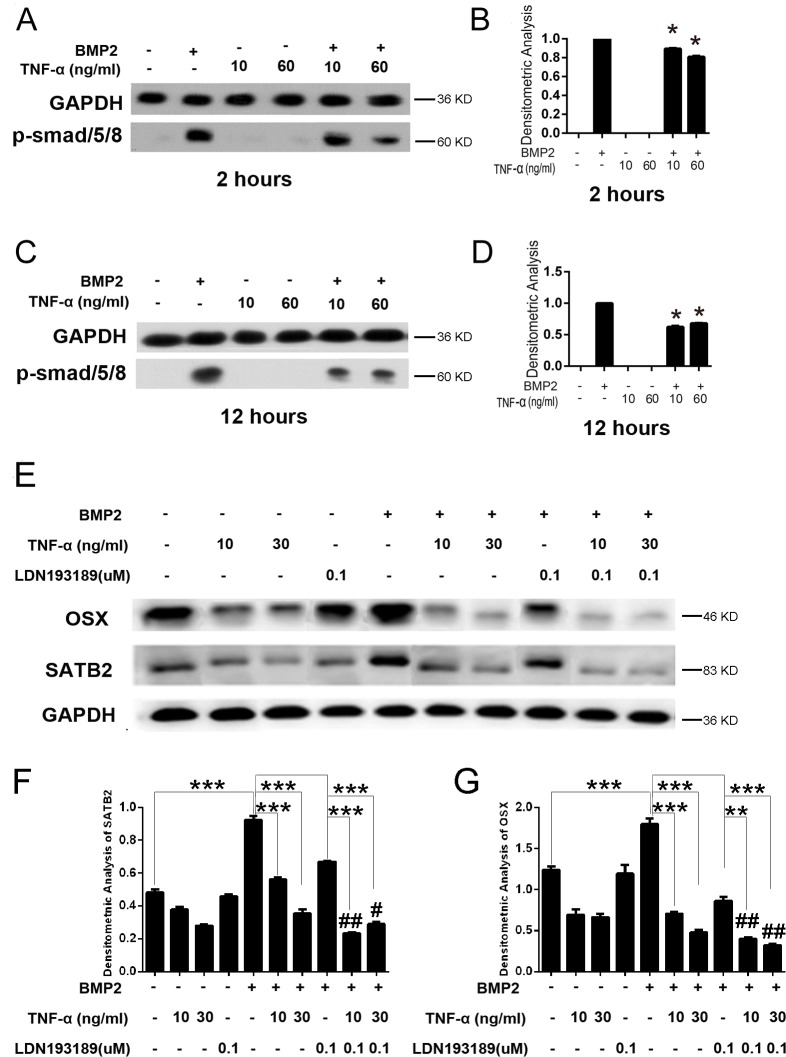
TNF-α inhibits SATB2 expression by inhibiting the smad1/5/8 signaling pathway **(A, C)** The C2C12 cells were treated with Adv-β-Gal (150 pfu/cell), Adv-BMP2 (150 pfu/cell), 10 ng/mL or 60 ng/mL of TNF-α for 2 or 12 h. Cell lysates were used to analyze p-smad1/5/8 activation. The GAPDH were used as loading controls. **(B, D)** Densitometric analysis of A and C respectively. **(E)** The C2C12 cells were treated with Adv-β-Gal (150 pfu/cell), Adv-BMP2 (150 pfu/cell), 10 ng/mL or 30 ng/mL of TNF-α, 0.1μM of LDN193189 for 72 h. The SATB2 and OSX expression levels were detected by western blotting. **(F, G)** Densitometric analysis of E. The data are presented as mean ± S.D. (*n* = 3; ^*^p < 0.05; ^**^p < 0.01; ^***^p < 0.001; ^#^*p* < 0.01; ^##^*p* < 0.001 compared with the related same treatment without LDN193189 administration). Uncropped western blot images corresponding to Figure 4(A, C and E) were shown in Supplementary Figure 2 (A, B and C respectively).

Bone morphogenetic proteins (BMPs), members of the transforming growth factor β superfamily (TGF-β) [25], strongly induce bone formation via triggering several critical osteogenic transcription factors such as Runx2 and Osx expression [26]. BMP-2 is a powerful differentiation factor that can recruit stem cells from surrounding muscle, bone marrow, or blood vessels to induce them into osteoblastic cells so as to create bone [21]. It is reported that Smad signaling is involved in BMP2-induced osteoblast differentiation in C2C12 cells [27, 28]. In facial skeletal development, Smad1/5, downstream of BMP2/4/7 signaling, has been shown to directly bind to the Satb2 promoter to induce SATB2 expression and function during osteoblast differentiation [21]. Therefore, we wonder whether TNF-α inhibit BMP2-induced SATB2 expression and osteoblast differentiation through repressing smad signaling especially smad1/5/8 activation. In this study, we tested the activation of smad1/5/8 and the expression levels of osteoblast marker genes after TNF-α and BMP2 treatment in C2C12 cells. The results showed that BMP2 outstandingly triggered smad1/5/8 phosphorylation but TNF-α significantly suppressed BMP2 induced smad1/5/8 activation in C2C12 cells (Figure 4A-4D). Moreover, the SATB2 expression was inhibited by TNF-α, which was significantly suppressed by further treatment with LDN193189, smad1/5/8 specific inhibitor, although it was suppressed by LDN193189 treatment alone as demonstrated by Figure 4E-4F. Furthermore, the expression of Osx was also repressed by TNF-α or LDN193189 and was further down-regulated by treatment with both TNF-α and LDN193189. (Figure 4E, 4G) These results suggest that TNF-α inhibits SATB2 gene expression and osteoblastogenesis by dampening smad1/5/8 signaling pathways.

### TNF-α inhibits SATB2 expression by activating MAPK-ERK signaling pathway

**Figure 5:**
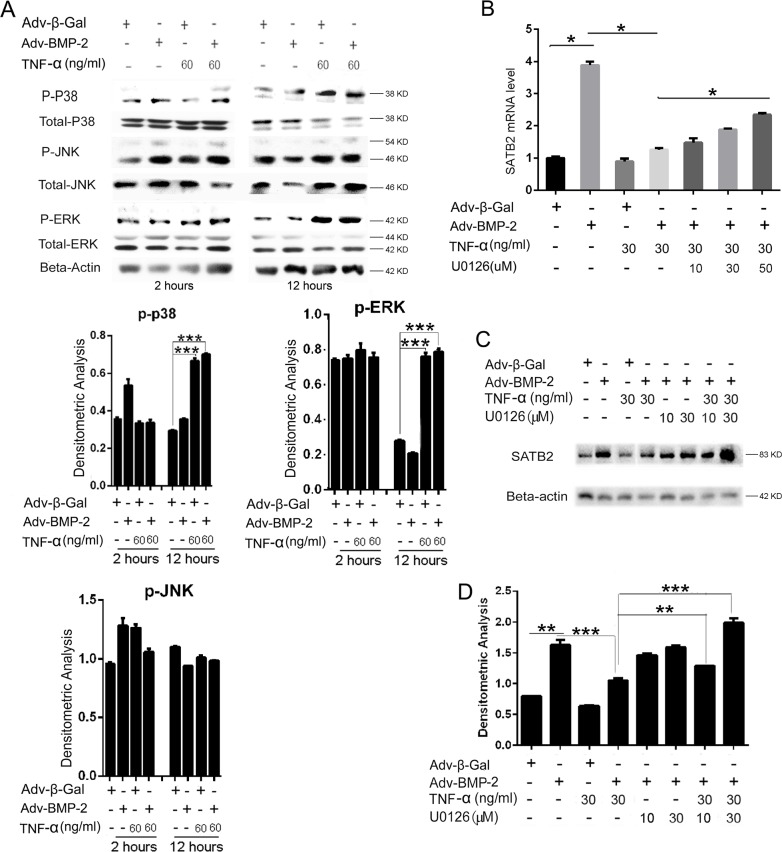
TNF-α inhibits SATB2 expression by activating the MAPK-ERK signaling pathway **(A)** The C2C12 cells were treated with Adv-β-Gal (150 pfu/cell), Adv-BMP2 (150 pfu/cell), or 60 ng/mL of TNF-α for 2 or 12 h. Cell lysates were used to analyze p-ERK, p-p38, and p-JNK activation. The total-ERK, total-p38, and total-JNK were used as loading controls, respectively. The relative densitometric analysis of p-p38, p-ERK and p-JNK were shown. These western blot images were uncropped. **(B, C)** The C2C12 cells were treated with Adv-β-Gal (150 pfu/cell), Adv-BMP2 (150 pfu/cell), 30 ng/mL of TNF-α or 10 μM to 50 μM of U0126 for 72 h. The SATB2 expression level was detected by real-time PCR (B) and western blotting (C). **(D)** Densitometric analysis of C. The data are presented as mean ± S.D. (*n* = 3; ^*^p < 0.05; ^**^p < 0.01; ^***^p < 0.001). Uncropped western blot images corresponding to Figure 5C was shown in Supplementary Figure 3.

The gene regulation function of TNF-α has two major downstream mediators: NF-κB and MAPK. We evaluated whether TNF-α can activate the MAPK pathway in C2C12 cells via the quantitative analysis of the activation of ERK1/2, p38, and JNK. We found that phosphor-ERK1/2 significantly increased after a 12 h treatment of TNF-α or a combination of TNF-α and Adv-BMP2, suggesting that TNF-α significantly activated the ERK pathway in C2C12 cells. Moreover, the addition of Adv-BMP2 was found to have no impact on the TNF-α -induced ERK signal activation (Figure 5A). Phosphor-p38 had a moderate increase, but no changes of phosphor-JNK were found (Figure 5A). Furthermore, we found that 50 μM of U0126, an ERK inhibitor, can totally block the ERK1/2 phosphorylation (Data not show), indicating that U0126 (50 μM) can block the TNF-α-induced ERK activation in C2C12 cells. Then, the C2C12 cells were treated with U0126 to abrogate the activation of the ERK signal by TNF-α, and the influence on SATB2 gene expression was evaluated. The results show that the SATB2 expression was inhibited by TNF-α, but was significantly rescued by 10 - 50 μM of U0126, as demonstrated by Figure 5B, 5C, 5D, besides, U0126 alone did not affect the expression of SATB2 as shown in Figure 5C-5D. These experimental results suggest that TNF-α inhibits SATB2 gene expression by promoting ERK signaling pathways.

### TNF-α inhibits SATB2 expression by activating NF-κB signaling pathway

**Figure 6:**
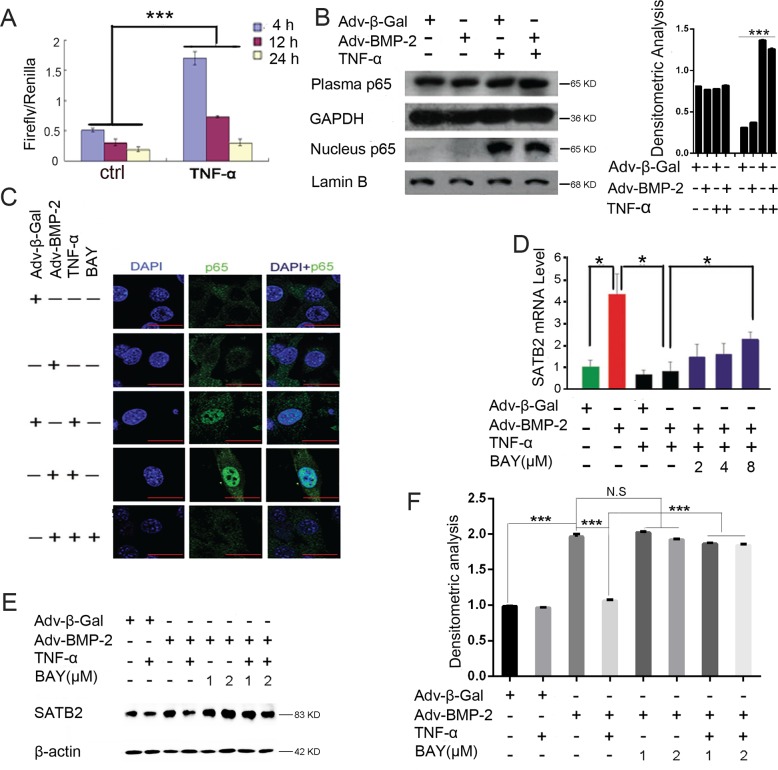
TNF-α inhibits SATB2 expression by activating NF-κB **(A)** The C2C12 cells were cotransfected with the pGL4.32[luc2P/NF-κB-RE/Hygro] vector and Rluc control reporter vector. 24 hours after transfection, the cells were treated with 40 ng/mL of TNF-α or PBS for 4h, 12h, 24h. Cell lysates were used to assess the luciferase activity. **(B)** The C2C12 cells were treated with Adv-β-Gal (150 pfu/cell), Adv-BMP2 (150 pfu/cell), or 60 ng/mL of TNF-α for 2 h. The cytoplasmic and nuclear proteins were isolated to assess the p65 level, uncropped western blot images and densitometric analysis of p65 were shown. **(C)** C2C12 cells were treated with Adv-β-Gal (150 pfu/cell), Adv-BMP2 (150 pfu/cell), 60 ng/mL of TNF-α or 8 μM of BAY11-7082 for 2 h and assayed to detect p65 protein (green) nuclear localization via a confocal microscopic method. DAPI (blue) was used for nuclear dyeing. (Bar=25μm) **(D, E, F)** The C2C12 cells were treated with Adv-β-Gal (150 pfu/cell), Adv-BMP2 (150 pfu/cell), 30 ng/mL of TNF-α, BAY11-7082 for 72 h. The SATB2 RNA expression levels (D) and protein levels (E, F) were examined. (F) The densitometric analysis for (E). The data are presented as mean ± S.D. (*n* = 3; ^*^p < 0.05; ^**^p < 0.01; ^***^p < 0.001; N.S means no significant difference). Uncropped western blot images corresponding to Figure 6E was shown in Supplementary Figure 4.

Aside from MAPK signaling pathways, NF-κB is another mediator of TNF-α response signaling. The activation of the NF-κB signaling pathway was reported to have a role in mediating the inhibition of osteoblast differentiation and *in vitro* bone formation. Thus, we examined whether NF-κB has a role in regulating SATB2 expression. The C2C12 cells were transfected with pGL4.32–NF-κB-RE–luciferase construct and the cell lysates were collected after 4, 12 and 24 h of TNF-α treatment; respectively. The results show that NF-κB luciferase activity was significantly increased by TNF-α treatment (Figure 6A). To determine whether TNF-α can induce P65 to translocate into the nucleus, we measured the nucleus and plasma p65 protein levels using the western blot analysis. Figure 6B shows that the nucleus p65 level was elevated after the treatment of TNF-α alone or the combined treatment of TNF-α and Adv-BMP-2 for 2 h. Subsequently, as shown in Figure 6C, the p65 protein (green) was found to exist in the nuclei of C2C12 cells after treatment with TNF-α or a combination of TNF-α and Adv-BMP2 for 2 h via a confocal microscopic analysis, and this effect could be blocked by an NF-κB specific inhibitor BAY11-7082 (8 μM). These results indicate that TNF-α can activate the NF-κB signaling activity in C2C12 cells and that the combined treatment with Adv-BMP2 does not attenuate or promote this activation effect. Moreover, the SATB2 mRNA (Figure 6D) and protein (Figures 6E-6F) expression are inhibited by TNF-α but is partly rescued by NF-κB inhibitor BAY11-7082. Besides, BAY11-7082 alone did not influence the expression of SATB2 as shown in Figure 6E-6F. The present results suggest that TNF-α inhibits the SATB2 gene expression by activating the NF-κB signaling pathway.

### TNF-α-induced NF-κB directly binds to SATB2 promoter to suppress its expression

**Figure 7:**
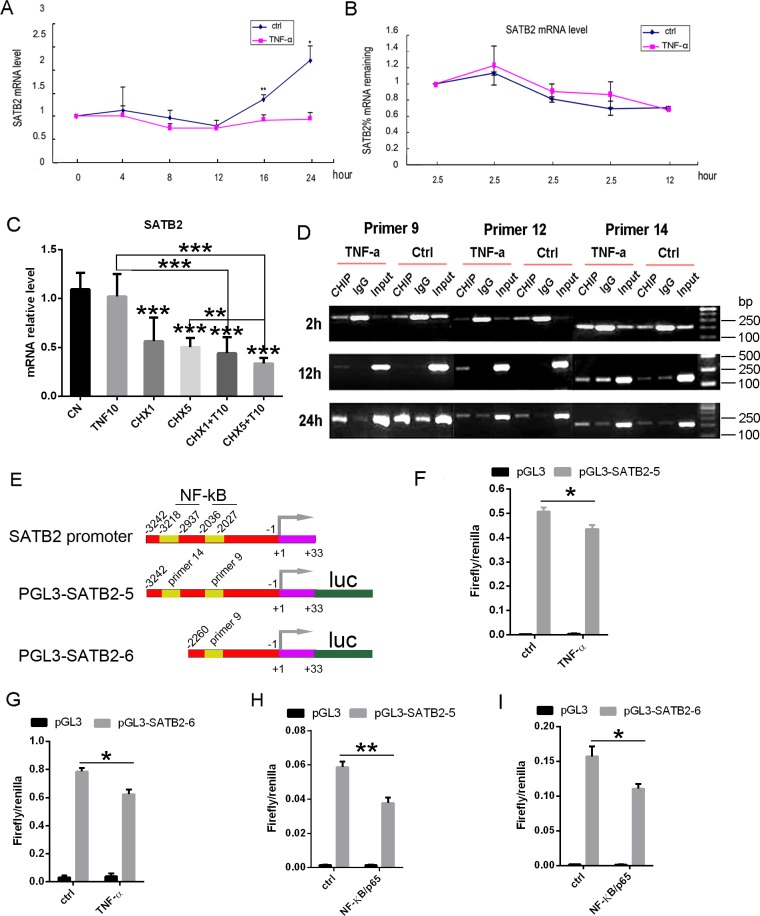
TNF-α induced NF-κB directly interacts with SATB2 gene promoter to inhibit its expression **(A)** Time course of TNF-α action. C2C12 cells were treated with BMP2 and with or without TNF-a (10ng/ml) for indicated times, SATB2 mRNA was measured by real time RT-PCR. **(B)** SATB2 mRNA stability was not changed by TNF-α. C2C12 cells were treated with BMP2 (150ng/ml) for one day and then treated with actinomycin D (0.5ug/ml) 2 hours before TNF-a (10ng/ml) were added, followed by detecting SATB2 mRNA relative levels at indicated time. **(C)** New protein synthesis is not required for TNF-α inhibition of SATB2 expression. Cycloheximide (CHX) (1 ug/ml, 5 ug/ml) was added 2 h prior to TNF-α (10 ng/ml, 20 ng/ml). SATB2 mRNA was measured 24 h after the addition of TNF-α. **(D)** C2C12 cells were treated with Adv-BMP2 and 10ng/ml TNF-α for indicated times and CHIP assay were performed with NF-kB/P65 antibody, followed by PCR with indicated primers. The gels have been run under the same experimental conditions. Uncropped images corresponding to Figure 7D were shown in Supplementary Figure 5. **(E)** NF-κB associate sites on SATB2 promoter and the schema of luciferase reporter gene plasmids construction. **(F, G, H, I)** Luciferase assay. Luciferase assay with plasmid pGL3-SATB2-5 (F) or pGL3-SATB2-6 (G) and treated with TNF-α. Luciferase assay with plasmid pGL3-SATB2-5 (H) or pGL3-SATB2-6 (I) and combined with NF-κB/p65 over-expression. The data are presented as mean ± S.D. (*n* =3; ^*^p < 0.05. ^**^p < 0.001, ^***^p < 0.0001).

To explore whether NF-κB inhibits SATB2 expression in a direct transcriptional way or not, we further studied the mechanism of TNF-α inhibition on SATB2 expression. Figure 7A shows that treatment with 10 ng/ml TNF-α inhibited SATB2 steady state mRNA. To determine whether TNF-α decreased SATB2 by destabilization of mRNA, C2C12 cells were treated with actinomycin D (0.5ug/ml) 2 hours prior to the addition of TNF-a (10ng/ml) to stop RNA synthesis. The SATB2 mRNA were measured by real time RT-PCR and the results show that TNF-α didn't decrease SATB2 mRNA levels (Figure 7B). Additionally, C2C12 cells were treated with cycloheximide 2 hours before TNF-α treatment to determine whether TNF-α action required new protein synthesis. Figure 7C shows that cycloheximide alone decreased SATB2 mRNA, although TNF-α further inhibited SATB2 mRNA level in the presence of cycloheximide treatment. This indicated that the effects of TNF-α on SATB2 expression are direct rather than requiring the indirect induction of a protein mediator. Furthermore, we used the software TFsearch to predict the association sites of NF-κB/p65 on SATB2 gene promoter, and based on the prediction results, several primers were designed to perform CHIP assay experiments. Figure 7D shows that under TNF-a treatment for 2, 12, 24 hours, primer 9, 12, 14 can all be associated with NF-kB/p65. To further confirm the association of NF-kB/p65 with SATB2 promoter, we constructed two luciferase reporter gene plasmids with pGL3-basic vector on the basic of CHIP assay results (Figure 7E). The plasmids pGL3-SATB2-5, pGL3-SATB2-6 and pGL3-basic vector were respectively cotransfected with pRL *Renilla* luciferase (Rluc) control reporter vector plasmids into C2C12 cells, followed by treatment with 40 ng/ml TNF-α or PBS for 4 hours. Then the firefly luciferase and renilla luciferase were detected. TNF-α treatment decreased the ratio of firefly and renilla luciferase (Figures 7F, 7G). Over-expression of NF-КB subunit p65 can also decrease the ratio of firefly and renilla luciferase without TNF-α treatment (Figure 7H, 7I). This suggested that NF-КB inhibits SATB2 expression through directly association with SATB2 promoter rather than indirect induction of a protein mediator.

## DISCUSSION

TNF-α is a critical inflammatory cytokine that induces bone loss in osteoporosis, rheumatoid arthritis [29], prosthetic loosening and other osteolytic diseases based on its osteoblastic differentiation inhibition and osteoclastic bone resorption stimulation, which leads to an ultimate imbalance in bone remodeling. The molecular mechanism of TNF-α inhibition of osteoblast differentiation still remains to be fully elucidated. In the current study, we are surprised to find that the expression of SATB2, a critical transcriptional node for osteoblast differentiation, is negatively associated with the levels of TNF-α in OVX-induced bone loss and IL-1β induced arthritis mice by immunohistochemistry. Then, we experimentally investigated the TNF-α inhibition of SATB2 during osteoblast differentiation. We proved that SATB2 expression is up-regulated by the BMP signal through time- and concentration-dependent manner by BMP2 stimulation and this up-regulation could be significantly inhibited by TNF-α in a concentration-dependent and durable manner. Besides, over-expression of SATB2 not only enhances ALP activity and osteoblastic differentiation marker genes OCN expression but also partly rescues TNF-α suppression of ALP activity and the expression of OCN during osteoblastogenesis. Furthermore, our present study indicated that TNF-α inhibited BMP2 induced smad signaling pathway so as to inhibit SATB2 expression and bone formation. These findings demonstrate a new mechanism for TNF-α inhibition of osteoblast differentiation and bone formation via the suppression of SATB2 expression.

The manner by which TNF-α inhibits SATB2 expression, either in a direct or indirect way is still to be explored. The inhibition of Runx2 and Osx expressions and the induction of Runx2 degradation by TNF-α have been discovered [4–6]. Recently, SATB2 was found to be in the downstream of Runx2 and Osx [30–33]. Runx2 indirectly activated SATB2 expression by suppressing miR-23-27a-24-2 [30], and miR31 [31], which in turn inhibits SATB2. Osx regulated SATB2 expression by directly binding to the SATB2 promoter [34]. These lines of evidence implicate an indirect inhibition of TNF-α on SATB2, which is mediated by Runx2 and Osx. TNF-α may first inhibit Runx2 and Osx, and the decrease of which may ultimately lead to SATB2 inhibition. However, some pieces of evidence indicate that SATB2 may, in turn, regulate Runx2 and Osx expressions, the real-time PCR assay revealed that the removal of SATB2 from the calvarial osteoblast modestly decreased Runx2 expression [10]. Furthermore, SATB2 was found to upregulate the Osx expression synergistically with Runx2 [12]. However, the relationship among TNF-α and SATB2, Runx2 and Osx remains to be further explored.

As signal mediators of TNF-α, the mitogen-activated protein kinase (MAPK) and nuclear factor–κB (NF-κB) signaling pathways and their role in the regulation of SATB2 expression were investigated. MAPK is a major signaling pathway that mediates TNF-α activity. Immediately after the TNF-α trimer was bound to TNFR1, TRAFs were activated, which transduces the signal to activate MAPK cascades, including ERK1/2, p38, and JNK [35]. Our results suggest that TNF-α significantly activated the ERK signal in C2C12 cells and modestly activated p38, but not JNK. Moreover, we found that the ERK-specific inhibitor U0126 significantly abrogated the TNF-α inhibition of SATB2. The blockage of the ERK signal was previously considered to have a synergistic effect with BMP action and ultimately facilitated the osteoblastic differentiation [36]. The chronic suppression of the MEK–ERK signaling pathway by the MEK inhibitor PD98059 was found to augment the osteoblast differentiation, as featured by the elevated expression of osteoblast specific marker genes in MC3T3-E1 and C2C12 cells [37]. This inhibitory effect of SATB2 by the ERK signal in the present study suggest that the ERK signal mediates the inhibition of TNF-α on SATB2 expression and partly explain the mechanism of ERK signaling in inhibiting osteoblast differentiation.

NF-κB has been known to be critical in regulating osteoclast differentiation and activation [1] and was recently recognized to be an important negative regulatory signaling pathway for osteoblast differentiation and bone formation [24, 38]. Thus, NF-κB was considered to be a critical node for coupling bone formation and bone resorption and ultimately the balance of bone remodeling. NF-κB was also considered to be an ideal therapeutic target for the prevention and treatment of osteoporosis and other inflammatory associated bone loss [2]. However, more investigations are still needed to elucidate the inhibition mechanism of NF-κB towards osteoblast differentiation and bone formation. In present study, we found that TNF-α significantly activated the NF-κB signaling pathway and the inhibitory effect of TNF-α on SATB2 expression can be partly abrogated by the NF-κB inhibitor BAY11-7082, suggesting that TNF-α inhibits SATB2 expression by activating the NF-κB signaling pathway. We further proved that TNF-α exerts no effects on SATB2 mRNA stability and it inhibits SATB2 expression independent of inducing protein mediators. In addition, the results of CHIP assay and luciferase reporter assay in current study indicate that NF-κB/p65 directly binds to 2kb-3kb upstream of SATB2 translational start sites, fine association sites remain to be explored. Another amazing point is that activation of NF-КB family members, especially RelA/p65 and p50, were physically interacted with STAT3 to serve significant roles in cellular activity [39–42] and STAT3 signaling is also critical for maintaining bone homeostasis and osteoblast differentiation [43–47]. Whether there is interaction between NF-КB and STAT3 in the modulation of NF-КB on SATB2 expression remains to be explored and uncovering this point sheds light on the accurate mechanisms of regulation of NF-КB on SATB2 expression.

**Figure 8:**
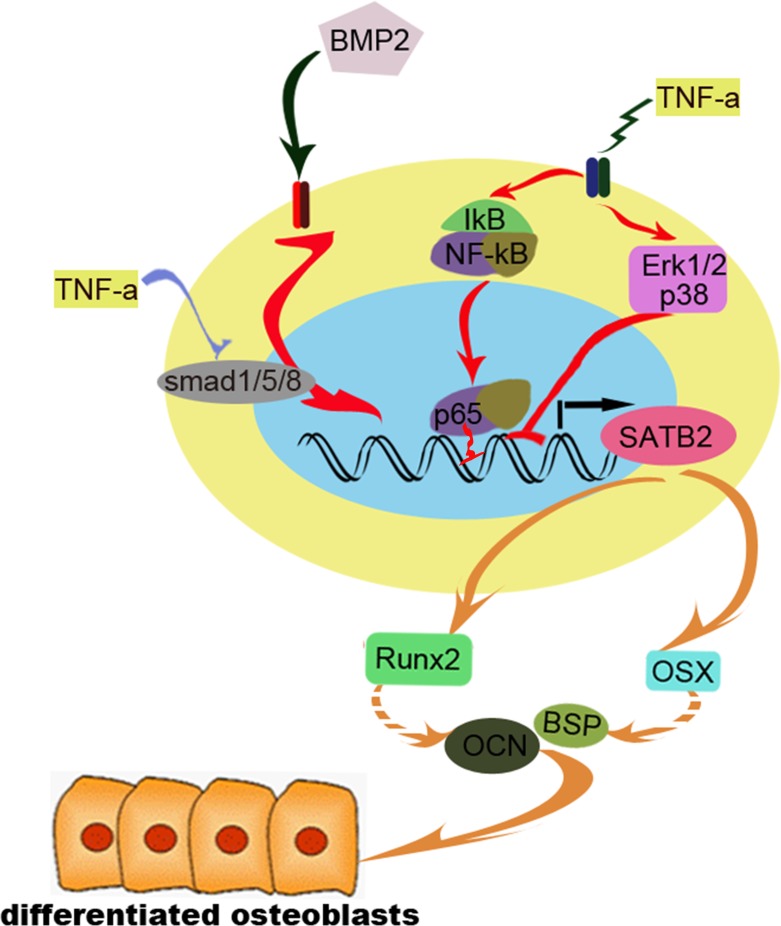
The diagram of our proposed working model BMP2 stimulates SATB2 expression and this up-regulation could be significantly inhibited by TNF-α, TNF-α inhibits BMP2 induced smad1/5/8 signaling, activates pERK1/2 and p38 signaling, in addition, TNF-α stimulates NF-κB signaling which results in translocation of p65 into nuclear and binds to SATB2 promoter to suppress SATB2 expression. Furthermore, the expression of osteoblast differentiation marker genes and osteoblastogenesis were inhibited.

In conclusion, the current study confirms that TNF-α inhibits SATB2 expression via BMP2/smad, MAPK and NF-κB signaling pathways, NF-κB/p65 directly interacts with SATB2 promoter to inhibit its expression, the decrease of SATB2 down-regulates the expression of osteoblast marker genes ALP, Osx and OCN and exerts inhibition toward osteoblast differentiation (Figure 8). The present study not only partly explains the mechanisms underlying the TNF-α-induced bone loss apart from the activation of osteoclasts, but also, it connects osteoblastic and osteoclastic regulation through Smad, MAPK, and NF-kB signaling pathway. Since ERK1/2 and NF-κB signals have been known to enhance bone resorption through stimulating osteoclasts differentiation and maturation, it is of great significance to design molecule drug for blocking MAPK and NF-κB signaling pathways to rescue bone loss by a bidirectional mechanism, via rescuing osteoblast inhibition and blocking osteoclast stimulation.

## MATERIALS AND METHODS

### Reagents

The C2C12 mesenchymal cells were obtained from the American Type Culture Collection (ATCC, Rockville, MD). The adenovirus-BMP2 (Adv-BMP2) and adenovirus-β-Gal (Adv-β-Gal) were provided by Dr. Jueren Lou (Institute of Biological Products, Shanghai, China). The Human TNF-α was purchased from PeproTech (300-01A; Rocky Hill, NJ). Real-time PCR was done via the ABI7900HT system using SYBR1Premix Ex Taq^TM^ (DRR041A; Takara, Dalian, China). The anti-SATB2 (1:1000; SATBA4B10), anti-Lamin B (1:500; ab151735) and anti-TNF-α (1:600; ab34674) antibodies were obtained from Abcam (Cambridge, MA). The anti-GAPDH antibody (1:10000; sc-32233) was obtained from Santa Cruz Biotechnology (Santa Cruz, CA). The anti-phospho-p44/42 ERK (1:1000; #4370) and anti-total-p44/42 ERK (1:1000; #4695), anti-phospho-p38 (1:1000; #4631) and anti-total-p38 (1:1000; #8690), and anti-phospho-JNK (1:1000; #4668) and anti-total-JNK (1:1000; #9252) antibodies, the anti-P65 (1:1000; #8442) and anti-beta-actin antibodies (1:10000; #3700) were purchased from Cell Signaling Technology (Danvers, MA).

### Ovariectomized mice model and IL-1β-induced arthritis mice model

C57BL/6J mice (8 weeks) were purchased from Shanghai SLAC Laboratory Animal Co., Ltd, and housed five per cage under standard conditions (12 h light/12 h dark cycle, 21°C controlled temperature). For ovariectomized mice model, the mice were sham operated (sham group, n=9) or ovariectomized (OVX group, n=9) as previously described [48], four weeks after surgery, the mice were sacrificed and the bones were analyzed. For IL-1β-induced arthritis mice model, mice were anesthetized with 250 mg/kg intraperitoneal tribromoethanol (Sigma-Aldrich, St. Louis, MO) and knees shaved with scissors, and the patellar ligament visualized through a small skin incision. Then, a total volume of 5μl containing recombinant IL-1β (401-ML; R&D) in PBS was injected through the infra patellar ligament into the left knee joint space using a 30G needle (n=9). The right knee was injected with 5 μl of PBS as control [49]. All animal procedures were done following standard procedures as required by our institution.

### Cell culture and osteoblastic differentiation

The C2C12 mesenchymal cells were obtained from American Type Culture Collection (ATCC, Rockville, MD, USA) and cultured in growth medium composed of DMEM (HyClone), 10% FBS (Gibco) and 1% penicillin–streptomycin at 37°C in the presence of 5% CO2. The C2C12 cells were plated at 1 × 10^5^ cells/well in a 12-well plate at day 0 and at the following day treated with Adv-β-Gal (150pfu/cell) and Adv-BMP2 (in the concentration indicated). For TNF-α inhibition, the C2C12 cells were treated with TNF-α (300-01A; ReproTech; Rocky Hill, NJ) at the indicated concentrations. For the NF-κB and ERK signal blockage, the cells were treated with BAY11-7082 (S2913; Selleck, USA) and U0126 (S1102; Selleck, USA), respectively, at the indicated concentration. The inhibitors were added 2 h prior to TNF-α treatment.

### Alkaline phosphatase (ALP) activity and staining

The C2C12 cells were rinsed twice with ice-cold PBS, scrapped from the dishes, and suspended in ddH_2_O. Then, the suspensions were freezed and thawed thrice. The ALP activity was determined at 405 nm using p-nitrophenyl phosphate (pNPP) (Sigma-Aldrich, St. Louis, MO) as substrate. 50 ul of the sample were mixed with 50 mL of pNPP (1 mg/mL) in 1 M of diethanolamine buffer containing 0.5 mM of MgCl_2_ (pH 9.8) and then incubated at 37°C for 15 min on a bench shaker. The reaction was stopped by the addition of 200 mL of 2 M NaOH per 200 mL of the reaction mixture. The total protein content was determined through the BCA method using the protein assay kit (PIERCE, Rockford, IL). The ALP activity was calculated as nanomoles of p-nitrophenol per minute per milligram of protein and presented as fold changes over the indicated control group at the respective time points. All experiments were conducted in triplicate. The ALP staining was performed according to manufacturer's instructions, briefly, the C2C12 cells were rinsed thrice with PBS and fixed with 4% paraformaldehyde for 10 min at 4°C. The fixed cells were soaked in 0.1% naphthol AS-MX phosphate (Sigma-Aldrich, St. Louis, MO) and 0.1% fast red violet LB salt (Sigma-Aldrich, St. Louis, MO) in 56 mM of 2-amino-2-methyl-1,3-propanediol (pH 9.9, Sigma-Aldrich, St. Louis, MO) for 10 min at room temperature (RT). Then, they were washed with PBS and observed under a digital camera [50].

### RNA harvest and real-time PCR

The total RNA of cells was isolated using TRIzol reagent (Invitrogen) according to the manufacturer's instructions. First-strand cDNA was synthesized from 1 μg of total RNA by incubating for 1 h at 42°C with Superscript III reverse transcriptase (Invitrogen, Mulgrave, Australia) following oligo(dT) priming. After reverse transcription reaction, qRT-PCR was performed by LightCycler480 system (Roche, Mannheim, Germany) using SYBR1Premix Ex TaqTM (Takara, Dalian, China) according to the manufacturer's instructions. Glyceraldehyde-3-phosphate dehydrogenase (GAPDH) was used as an internal control. Data were analyzed using the comparison Ct (2^-ΔΔCt^) method [51] and expressed as fold change compared to respective control. Each sample was analyzed in triplicate. The primer sequences used in this study were as follows:

GAPDH: forward,5′-GACTTCAACAGCAACTCC CAC-3′; reverse, 5′-TCCACCACCCTGTTGCTGTA-3′; Collagen I (Col I): forward, 5′-GAGCTGGTGTAA TGGGTCCT-3′; reverse, 5′-G AGACCCAGGAAGACC TCTG-3′; *bsp*: forward, 5′-AGTGTGGAAAGTGTGG CGT T3′; *Ocn:* forward, 5′-AAGCAGGAGGGCAATA AGGT-3′; reverse, 5′-TTTGTAGGC GGTCTTCAAGC-3′; and *SATB2*: forward, 5′-GCCGTGGGAGGTTTGATG ATT-3’; reverse, 5′-ACCAAGACGAACTCA- GCGTG-3′.

### Construction of lentiviral SATB2 stable transfectant of C2C12 cells

The lentiviral vector LV5-homo-SATB2 that contain human SATB2 gene or control lentiviral vector LV5NC were constructed by Genepharma Company (Shanghai, China). Stable SATB2 transfectants were selected by puromycin (Sigma-Aldrich) 72 h after lentivirus infection. The stable transfected cells were used for further studies.

### Confocal microscopic assay for p65 localization

The C2C12 cells were treated with Adv-β-Gal, Adv-BMP2, TNF-α and BAY11-7082 (8 μM), washed twice with PBS, and fixed with 4% paraformaldehyde for 20 min. The fixed cells were incubated with the primary anti-p65 antibody (1:100) overnight in 4°C and with the fluorescent secondary antibody for 1h at RT. 4′,6-diamidino-2-phenylindole (DAPI) was used to stain the DNA nucleus of the cells. The stained cells were then analyzed via Leica TCS SP5 confocal laser scanning microscopy (Leica, Wetzlar, Germany).

### CHIP assay (chromatin immunoprecipitation assay)

The ChIP assay kit (EZ ChIP™ Chromatin Immunoprecipitation Kit Catalog # 17-371) was obtained from Millipore. CHIP assays were performed according to the manufacture's description with some modifications. Briefly, on the first day the C2C12 mesenchymal cells were cultured in DMEM supplemented with 10% FBS and 50 U/mL penicillin, and 50 mg/mL streptomycin (all from Hyclone, Logan, UT) in 10cm dish and stimulated with adv-BMP2 (150 pfu/cell) on the second day when the cells were 70-80% confluence. 24 hours later, TNF-α (40ng/ml) was added to the medium. Cells were washed with ice-cold PBS twice after 2, 12 and 24 hours of the stimulation with TNF-α. 1% Formaldehyde (Invitrogen) was used to cross-link the cells for 10 min, and cross-linking was quenched with glycine. Cells were harvested and rinsed with PBS, and cell pellets were resuspended in 1 ml of SDS lysis buffer. After sonication, 100 ul of the sheared chromatin was diluted to 1ml with IP dilution buffer for each immunoprecipitation. The chromatin solution was pre-cleared with 30 μl of protein G-agarose beads at 4°C for 1 h. The precleared chromatin was collected and incubated at 4°C overnight with 10 μl of anti-p65/NF-kB antibody or 1μg IgG as a negative control. The immune complexes were precipitated with 60 μl of protein G-agarose beads at 4°C for 1 h. After washes, the antibody-protein-DNA immunocomplexes were eluted twice with 100 μl of elution buffer. Formaldehyde cross-linking was reversed by heating at 65°C overnight with the addition of 5M NaCl. All of the samples were digested with RNase A and proteinase K. The DNA was purified using spin columns and analyzed by PCR gel. The primer sets used for amplification of Satb2 promoter regions were designed with primer 5 randomly or based on the software TFsearch prediction. The primer sets are, primer9: forward, 5′- TCAGCCCCTGGAACCTCAAA-3′; reverse, 5′- CTACCAAGCAAGTGGACAGC-3′; primer12: forward, 5′-AGAAAAGCAGTCTAGCAAGCG-3′; reverse, 5′-TTCAAAACTCCTCCTCACCC-3′; primer14: forward, 5′-CCAGTGAGGTTAGCGAAGAG-3′; reverse, 5′-CCAATGTCCACGAACTAAGAAG- 3′.

### Luciferase reporter plasmid construction

The progressive deletion fragments of the Satb2 promoter region (-3242/+33) were generated by PCR using mouse genomic DNA as a template and the resulting PCR products were digested with Kpn I and Hind III and inserted into the firefly luciferase reporter vector pGL-3 basic vector (Promega, Madison, WI). Primers were designed using the primer 5 and the primer sequences are as follows: SATB2-5-Kpn I forward; 5’-3’ cgGGGTACCAACCGATTAAACAATACTGC; SATB2-6-Kpn I forward; 5’-3’ cgGGGTACCTAC CAAGCAAGTGGACAGCA; SATB2 reverse 5’-3’ cccaagcttGGTTCGGAGATGGTTGTTATG.

### Transfection and Luciferase assay

The day before transfection, 1 × 10^5^ cells in 1 ml of DMEM with 10% FBS and 50 mg/mL streptomycin were seeded in each well of 12-well plates, so that cells could be 80-90% confluent at the time of transfection. Transfection was performed according to the manufacturer's protocol (Invitrogen, USA). For each transfection sample, reactions were prepared as follows: Dilute DNA (plasmids with genes of interest and plasmids with Renilla for reference) in 100 μl of Opti-MEM® I Reduced Serum Medium and mix gently. Mix Lipofectamine™ 2000 gently before use, then dilute 3.5 ul in 100 μl of Opti-MEM® I Medium. After the 5 minute incubation at room temperature, combine the diluted DNA with diluted Lipofectamine™ 2000 (total volume = 200 μl per well) and mix gently, then incubate for 20 minutes at room temperature. Add the 200 μl of complexes to each well containing cells and medium. Mix gently by rocking the plate back and forth followed by incubating cells at 37°C in a CO2 incubator. The plasmids pGL3 basic vector, pGL3-SATB2-5 or pGL3-SATB2-6 were cotransfected with the pRL *Renilla* luciferase (Rluc) control reporter vector plasmids and with or without NF-kB/P65 into C2C12 cells. After transfection, cells were incubated in a CO2 incubator at 37°C for 12 hours and then 150 ng/ml of human recombinant BMP2 (R&D) were added in each well. 24 hours after transfection, 40 ng/ml TNF-α or PBS were added and cell extracts were harvested 4 or 12 h later for luciferase assay. The pGL4.32 [luc2P/NF-κB-RE/Hygro] vector plasmids were transfected into C2C12 cells to determine the NF-κB activity. The pRL *Renilla* luciferase (Rluc) control reporter vector plasmids were cotransfected with pGL4.32 [luc2P/NF-κB-RE/Hygro] vector plasmids as an inner control. These two plasmids were both purchased from Promega (Madison, WI) and transfected into C2C12 using Lipo2000 (Invitrogen). The C2C12 cells were seeded at 0.5 × 10^5^ per well in a 24-well plate in complete medium and stimulated with TNF-α or PBS for 24 h after plasmid transfection. The cells were harvested 2, 12 and 24 hours later, and their luciferase activities measured using a dual luciferase system (Promega, Madison, WI) according to the manufacture's description.

### Statistical analysis

All data are expressed as mean ± S.D. Student's t-test for the comparison between two groups or one-way ANOVA followed by Tukey's multiple-comparison posttest for multiple groups was performed. P< 0.05 was considered to be significant.

## SUPPLEMENTARY MATERIALS FIGURES AND TABLES


