# Supplementary material for: TNF-α inhibits SATB2 expression and osteoblast differentiation through NF-κB and MAPK pathways
Source: Oncotarget. 2017 Dec 18;9(4):4833–50. doi: 10.18632/oncotarget.23373 (PMC5797016; doi:10.18632/oncotarget.23373)
Supplement: Supplementary file 1 [file oncotarget-09-4833-s001.pdf]

TNF-α inhibits SATB2 expression and osteoblast differentiation through NF-κB and MAPK pathways

SUPPLEMENTARY MATERIALS

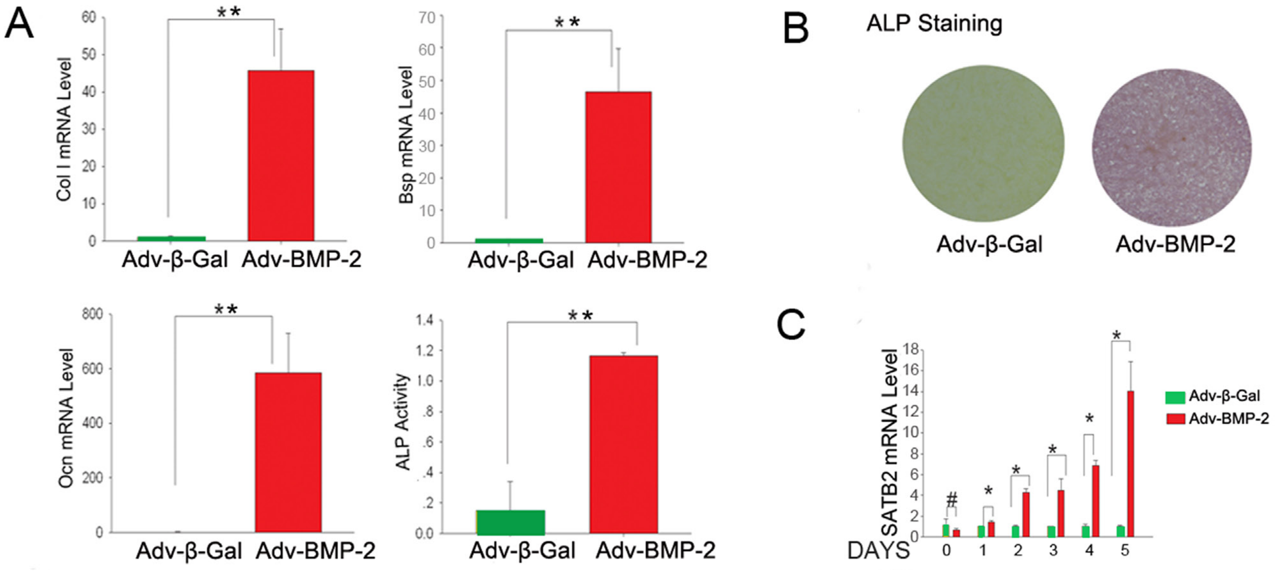

Supplementary Figure 1: Adv-BMP2 induces C2C12 cells to differentiate into osteoblasts and promotes SATB2 expression. The C2C12 cells were treated with Adv-BMP2 (150 pfu/cell) or Adv-β-Gal (150 pfu/cell) for five days **(A)** The *Col I, Bsp*, and *Ocn* mRNA levels were examined. The ALP activity was normalized to the Adv-β-Gal group, and **(B)** ALP staining was performed, **(C)** The C2C12 cells were treated with Adv-BMP2 (150 pfu/cell) or Adv-β-Gal (150 pfu/cell) for various time points and the *SATB2* gene expressions were assessed via real-time PCR. The fold change was calculated as follows: (relative *Col I*, *Bsp*, *Ocn, SATB2* levels in Adv-BMP2-treated sample) /(relative *Col I*, *Bsp*, and *Ocn* levels in Adv-β-Gal-treated sample), the data were presented as mean ± S.D. ($n = 3$; $^{*}p < 0.05$; $^{**}p < 0.01$).

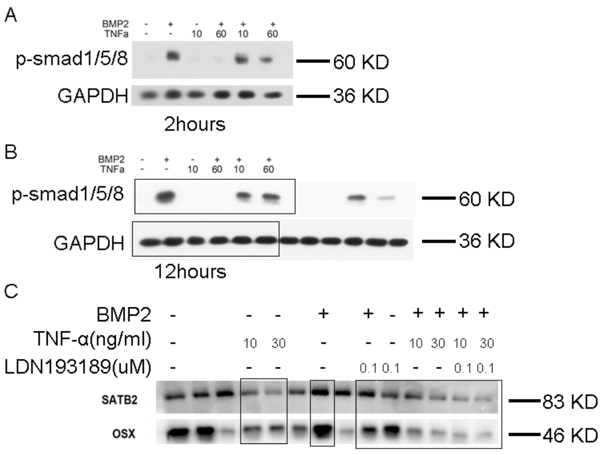

Supplementary Figure 2: TNF-α inhibits SATB2 expression by inhibiting the smad1/5/8 signaling pathway. Uncropped western blot images corresponding to Figure 4(A), Figure 4(C) and Figure 4(E).

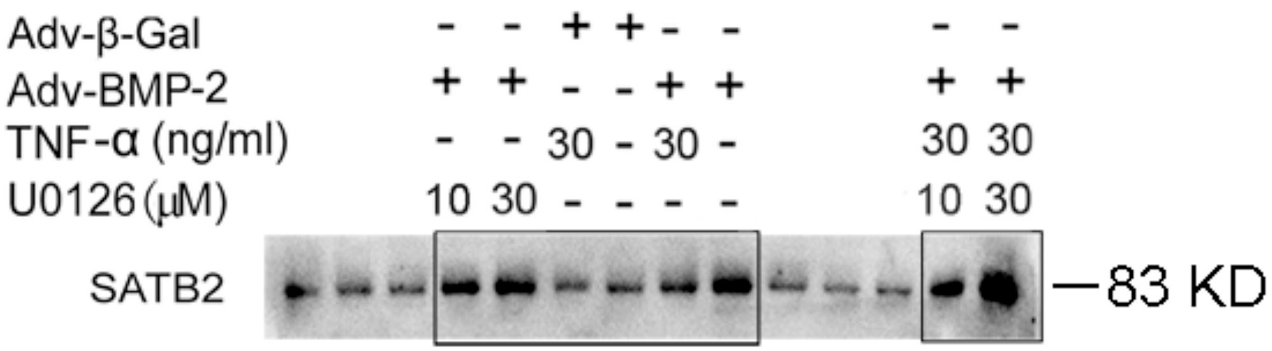

Supplementary Figure 3: TNF-α inhibits SATB2 expression by activating the MAPK signaling pathway. Uncropped western blot images corresponding to Figure 5C.

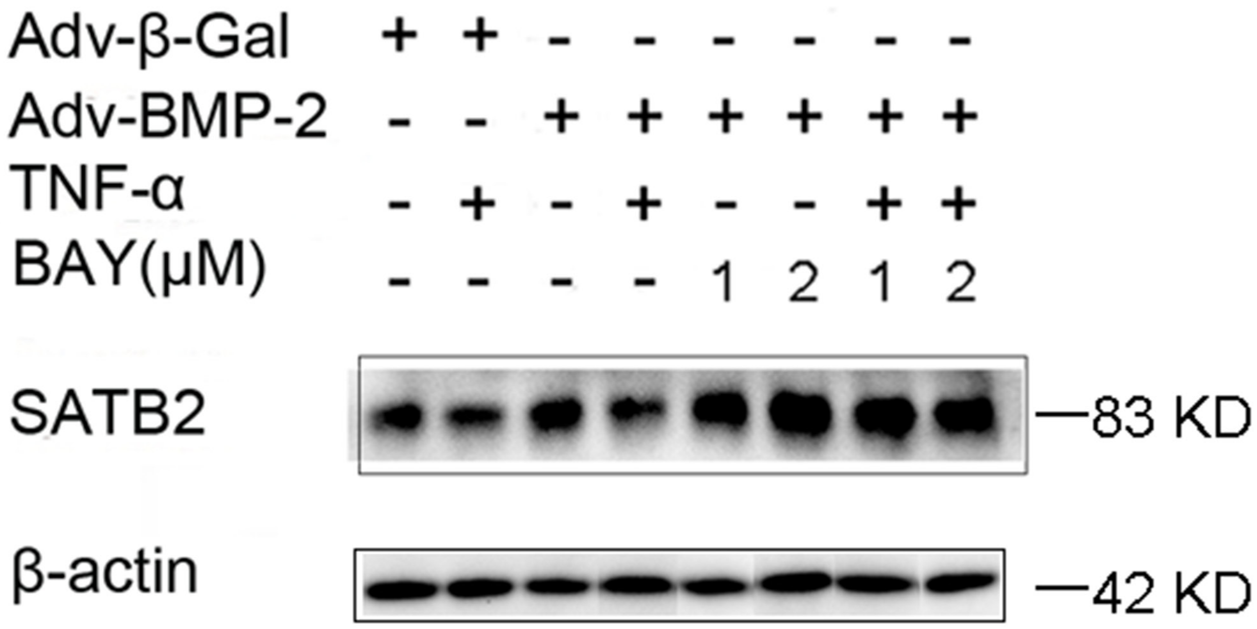

Supplementary Figure 4: TNF-α inhibits SATB2 expression by activating NF-κB. Uncropped western blot images corresponding to Figure 6E.

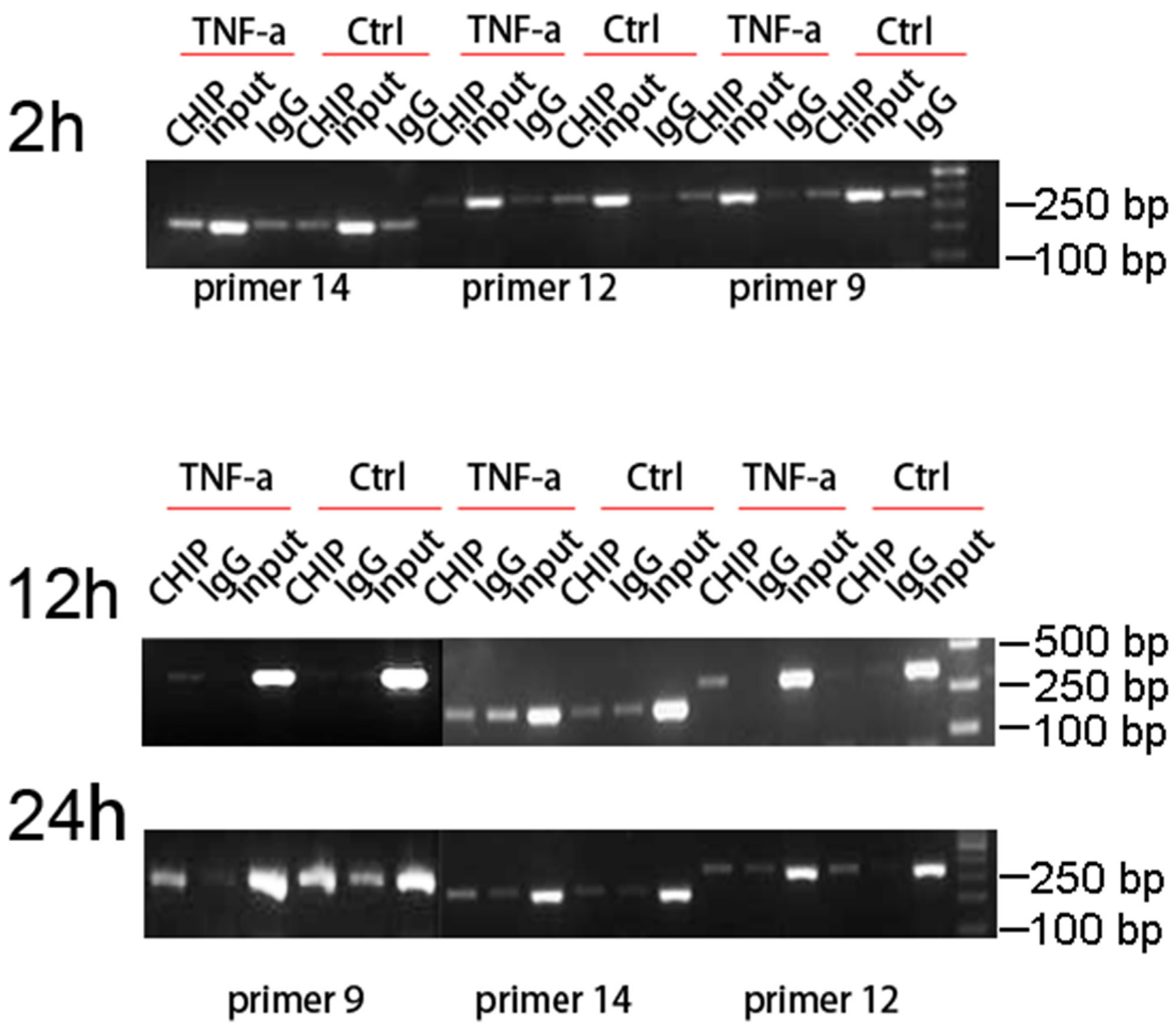

Supplementary Figure 5: TNF-α induced NF-κB directly interacts with SATB2 gene promoter to inhibit its expression. Uncropped RT-PCR images corresponding to Figure 7D.
